# Predictive Value of Individual Behavioral Risk Factors for New Mood‐Related Psychiatric Disorder After Diagnosis of Cancer

**DOI:** 10.1002/pon.70046

**Published:** 2024-12-19

**Authors:** Seiichi Villalona, Carlos Chavez Perez, E. Paul Wileyto, Samuel Takvorian, Peter Gabriel, Abigail Doucette, Daniel Blumenthal, Robert Schnoll

**Affiliations:** ^1^ Department of Medicine University of Pennsylvania Perelman School of Medicine Philadelphia Pennsylvania USA; ^2^ Department of Internal Medicine Hospital of the University of Pennsylvania Philadelphia Pennsylvania USA; ^3^ Biostatistics University of Pennsylvania Philadelphia Pennsylvania USA; ^4^ Division of Hematology & Oncology Department of Medicine University of Pennsylvania Perelman School of Medicine Philadelphia Pennsylvania USA; ^5^ Abramson Cancer Center University of Pennsylvania Philadelphia Pennsylvania USA; ^6^ Department of Biostatistics University of Pennsylvania Perelman School of Medicine Philadelphia Pennsylvania USA; ^7^ Department of Psychiatry University of Pennsylvania Perelman School of Medicine Philadelphia Pennsylvania USA

**Keywords:** anxiety, depression, HPV‐associated cancers, Psycho‐oncology, tobacco use

## Abstract

**Objective:**

The diagnosis of a mood‐related psychiatric disorder (MRPD) among patients with cancer has been associated with decreased quality of life and lower cancer survival. This study aimed to understand the risk of a new MRPD after cancer diagnosis by individual risk behaviors, with a specific focus on tobacco use and the presence of a human papillomavirus (HPV)‐associated cancer.

**Methods:**

Single‐center retrospective cohort study of 11,712 patients diagnosed with cancer between 2009 and 2020. We identified predictors of a new MRPD after cancer diagnosis using a time‐to‐event analysis and Cox proportional hazards model including demographics, disease characteristics, and tobacco use and HPV‐associated tumors.

**Results:**

Univariate analyses revealed lower hazard ratios (HRs) of a new MRPD among individuals that identified as Asian/Pacific Islanders and among the older age groups (> 51 years). Univariate analyses additionally demonstrated higher HRs of MRPD among females; sexual minorities; former and current smokers; individuals with HPV‐associated cancers; and individuals diagnosed at later stages. These relationships were observed in the multivariate model when adjusting for covariates. Shorter time‐to‐MRPD was observed when stratifying by individual behavioral risk factors, with active smokers and individuals with an HPV‐associated cancer being at the highest risk.

**Conclusions:**

Individual behavioral risk factors increase risk of new MRPD after being diagnosed with cancer. These findings build on past studies by linking tobacco use and HPV‐associated cancers with MRPD risk in oncology and can be used to identify patients at risk of developing new MRPDs post‐cancer diagnosis and engaging them in treatment.

## 1 Background

Patients with cancer report a higher prevalence of mood related psychiatric disorders (MRPDs) relative to the general population [1, 2]. Across multiple cancers, comorbidity of a MRPD has been associated with poorer quality of life, decreased treatment adherence, early mortality, and lower overall survival [3, 4, 5, 6]. The effect of depression on increased mortality has been attributed to neuroendocrine‐immunological dysregulation, decreased physical activity, side effects from treatment, and increased consumption of nicotine and alcohol [4, 7]. Research suggests that the increased risk of a psychiatric diagnosis may develop long before a cancer diagnosis due to multiple biopsychosocial processes including history of substance abuse and decreased quality of life pre‐diagnosis [8]. Lu et al. (2016) found that depressive symptoms begin upwards of 5 years before the first hospitalization for cancer, hinting at a need for early identification and treatment of psychiatric symptoms when a patient is first diagnosed with cancer [8].

Among the cancers associated with an individual behavioral risk factor, the associations of depression and anxiety has been extensively studied among patients with lung cancer [6, 9, 10, 11]. The role of stigma and self‐blame has also been explored among cancer patients, particularly with regard to the presence of tobacco use [9]. Patients with human papillomavirus (HPV)‐associated cancers may also be at heightened risk for adverse psychiatric outcomes given the association of their malignancies with sexual activity. HPV is the most common sexually transmitted infection (STI) in the United States and is associated with a majority of cancers involving the oropharynx, anal canal, cervix, vagina, vulva, and penis [12]. HPV positivity has been associated with increased anxiety and depressive symptoms among patients without a cancer diagnosis [13, 14]. Most of the limited studies that have assessed MRPDs among patients with HPV‐associated cancers have primarily focused on either oropharyngeal or cervical cancers [15, 16, 17, 18, 19]. Among patients with HPV‐associated anogenital and other gynecologic cancers (vulvar or vaginal), prior research has identified the sentiments of shame, embarrassment and guilt that come from being diagnosed with these cancer types [20, 21, 22].

The limited data on assessing comorbid anxiety and depressive symptoms among patients with HPV‐associated cancers has been mixed in terms of the incidence (i.e. present at the time of cancer diagnosis vs. developing them de novo post‐diagnosis) and the persistence of these symptoms [15, 23, 24]. To the best of our knowledge, there is no previous study that has examined HPV‐association (regardless of anatomic site) with developing de novo MRPDs after cancer diagnosis. This study aimed to address this gap within the literature by assessing demographic and disease‐related characteristics, as well as tobacco use and having an HPV‐associated tumor, as predictors of a new MRPD. This study specifically tested the hypothesis that individuals with a history of smoking and/or HPV associated cancer experience higher odds of developing a new MRPD post‐cancer diagnosis.

## 2 Methods

### 2.1 Participants and Procedures

This single‐center retrospective cohort study was conducted among a cohort of patients receiving care in the University of Pennsylvania Health System and diagnosed with breast, prostate, colon, cancers lung, oropharyngeal, anorectal, gynecologic (cervical, vaginal, and vulvar), or penile cancers between 01/01/2018–12/31/2019. These cancers encompassed the four most common cancers among men and women, as well as those highly associated with individual behavioral risk factors. Inclusion criteria for the study included age > 18 years at cancer diagnosis, having at least one return patient visit after diagnosis during the study period, and have smoking status assessed within 30 days of cancer diagnosis. Cases were excluded if they had a documented MRPD prior to their cancer diagnosis and if they had missing data for any other independent variables. Data were extracted from the cancer registry at the Abramson Cancer Center as well as the electronic medical record used by the University of Pennsylvania Health System. This study was approved as an exempt study by the Institutional Review Board at the University of Pennsylvania (Protocol #853861). Informed consent was not obtained from individual participants since this study employed a retrospective observational design.

### 2.2 Measures

Independent variables collected for the study included tobacco use history and HPV‐association. Tobacco use history was determined using the self‐reported data available in the electronic medical record (never, former, current). Individuals were grouped into the former or current smoker groups regardless of dose, as this information was not available within the electronic medical records. Using cancer diagnosis, we classified HPV‐associated cancers using a combination of anatomic sites under the International Classification of Diseases for Oncology (ICD‐O)‐3 and histological codes for squamous cell carcinoma (SCC) (8050‐8084; 8120‐8131) [25]. HPV‐associated cancers were oropharyngeal, anorectal, gynecologic (cervical, vaginal, and vulvar) and penile cancers; non‐HPV associated cancers were tumors in the same anatomic location of different histologic type (i.e. adenocarcinoma or neuroendocrine) and tumors from any other anatomic site. While HPV status was not molecularly verified, we used the same classification methods previously used by cancer registry‐based studies with HPV‐associated cancers [26, 27, 28]. The control group consisted of patients with prostate, breast, and colorectal cancers since they are considered to be less associated with the individual behavioral risk factors of HPV or tobacco use. Covariates included in the study were age at diagnosis, sex, self‐reported race/ethnicity, self‐reported sexual orientation, cancer diagnosis, disease stage at diagnosis, and treatment modality.

### 2.3 Outcomes

The primary outcome for this study was development of a new MRPD post‐cancer diagnosis. The MRPDs included were: adjustment disorder; generalized anxiety disorder; bipolar disorder (currently depressive episode); major depressive disorder; and dysthymic disorder.

### 2.4 Statistical Analyses

Bivariate associations were explored using χ^2^ tests. Univariate relationships for time‐to‐diagnosis of a new MRPD post‐cancer diagnosis were assessed with Cox proportional hazards regression modeling. Multivariate analyses were conducted while adjusting for all other covariates. Kaplan–Meier curves estimating time‐to‐MRPD were generated from the multivariate model. The relationships between individual behavioral risk factors (tobacco use and HPV‐associated cancers) and new MRPD were assessed with never smokers and non‐HPV associated cancers as reference groups, respectively.

## 3 Results

As shown in Table 1, the average age of the sample was 63 years (SD = 12 years). The sample (*N* = 11,712) was predominantly comprised of females (58.5%), individuals that identified as non‐Hispanic White (71.7%), heterosexual (66.6%), and those diagnosed at disease stages I or II (70.6%). A majority of the sample received other treatment modalities (i.e. chemotherapy, immunotherapy, surgery, radiation, etc.) aside from diagnostic biopsy alone or hormonal monotherapy. Approximately, half of the sample had prior or current tobacco use history (49.4%) and a small proportion of cases (*n* = 328, 2.8%) in the sample consisted of those diagnosed with an HPV‐associated cancer. The three most represented cancer diagnoses in the sample included breast (*n* = 4650; 39.7%), prostate (*n* = 3017; 25.8%), and lung (2470; 21.1%).

**TABLE 1:** Characteristics of study sample (*N* = 11,712).

Characteristic	Total; N (%)	Individuals without a new mood related disorder after cancer diagnosis;	Individuals with a new mood related disorder after cancer diagnosis;	*p*
		N (%)	N (%)	
Overall	11,712 (100)	8679 (74.1)	3033 (25.9)	
Age at diagnosis				< 0.001
< 50	1984 (16.9)	1326 (15.3)	658 (21.7)	
51–64	4381 (37.4)	3189 (36.7)	1192 (39.3)	
> 65	5347 (45.7)	4164 (48.0)	1183 (39.0)	
Sex				< 0.001
Male	4856 (41.5)	3912 (45.1)	944 (31.1)	
Female	6856 (58.5)	4767 (54.9)	2089 (68.9)	
Race/Ethnicity				< 0.001
Non‐Hispanic White	8399 (71.7)	6179 (71.2)	2220 (73.2)	
Other^a^	2955 (25.2)	2199 (25.3)	756 (24.9)	
Asian/Pacific Islander	358 (3.1)	301 (3.5)	57 (1.9)	
Sexual orientation				< 0.001
Heterosexual/Straight	7801 (66.6)	5541 (63.8)	2260 (74.5)	
Homosexual/Lesbian, Gay, or Bisexual	154 (1.3)	85 (1.0)	69 (2.3)	
Other/Unknown/Undisclosed	3757 (32.1)	3053 (35.2)	704 (23.2)	
Stage at diagnosis				< 0.001
0	910 (7.8)	689 (7.9)	221 (7.3)	
I	4172 (35.6)	3131 (36.1)	1041 (34.3)	
II	3182 (27.2)	2454 (28.3)	728 (24.0)	
III	2116 (18.1)	1530 (17.6)	586 (19.3)	
IV	1332 (11.4)	875 (10.1)	457 (15.1)	
Treatment modality				< 0.001
Diagnostic biopsy only	532 (4.5)	449 (5.2)	83 (2.7)	
Biopsy & hormonal Monotherapy	128 (1.1)	103 (1.2)	25 (0.8)	
All other treatment	11,052 (94.4)	8127 (93.6)	2925 (96.4)	
Modalities				
Tobacco use				< 0.001
Never smoker	5929 (50.6)	4605 (53.1)	1324 (43.7)	
Former smoker	4504 (38.5)	3249 (37.4)	1255 (41.4)	
Current smoker	1279 (10.9)	825 (9.5)	454 (15.0)	
Human papillomavirus (HPV) status				< 0.001
Non‐HPV associated	11,384 (97.2)	8468 (97.6)	2916 (96.1)	
HPV Associated	328 (2.8)	211 (2.4)	117 (3.9)	
Cancer type				< 0.001
Breast	4650 (39.7)	3326 (38.3)	1324 (43.7)	
Prostate	3017 (25.8)	2544 (29.3)	473 (15.6)	
Gastrointestinal (Pancreatic/Colon)	848 (7.2)	623 (7.2)	225 (7.4)	
Lung	2470 (21.1)	1685 (19.4)	785 (25.9)	
Gynecological (Cervical/Vaginal/Vulvar)	148 (1.26)	95 (1.1)	53 (1.7)	
Penile	14 (0.1)	11 (0.1)	3 (0.1)	
Oropharyngeal	57 (0.5)	35 (0.4)	22 (0.7)	
Anorectal	508 (1.3)	360 (4.1)	148 (4.9)	

^a^ Other race included NH Black, Hispanic (all Races), and Multiple Races.

3033 individuals (26%) in the study sample developed a clinically significant mood‐related psychiatric disorder (MRPD) after being diagnosed with cancer. Bivariate associations were observed by age, sex, race, sexual orientation, stage at diagnosis, tobacco use history and HPV association. Relative to the entire study sample, higher proportions of those diagnosed with new MRPDs were comprised of individuals who were younger (< 50 years) (21.7% vs. 16.9%, *p* < 0.001); female (68.9% vs. 58.5%, *p* < 0.001); identified as a sexual minority (i.e. identified as homosexual/Lesbian, Gay, or Bisexual) (2.3% vs. 1.3%, *p* < 0.001); diagnosed at stage III or IV disease (19.3% and 15.1% vs. 18.1% and 11.4%, respectively, *p* < 0.001); reported being a former or current smoker at cancer diagnosis (41.4% and 15% vs. 38.5% and 10.9%, respectively, *p* < 0.001); and those diagnosed with an HPV‐associated cancer (3.9% vs. 2.8%, *p* < 0.001). Relative to the study sample, lower proportions of those diagnosed with new MRPDs identified as being Asian/Pacific Islanders (1.9% vs. 3.1%, *p* < 0.001).

### 3.1 New Mood‐Related Psychiatric Disorder Post‐Cancer Diagnosis

Univariate analyses revealed lower hazard ratios (HRs) of a new MRPD among individuals that identified as Asian/Pacific Islanders and among the older age groups (> 51 years) (Table 2). Univariate analyses additionally demonstrated higher HRs of MRPD among females; those that identified as sexual minorities; former and current smokers at time of cancer diagnosis; individuals with HPV‐associated cancers; individuals diagnosed at later disease stages (III and IV); and those who received all other treatment modalities other than hormonal monotherapy (Table 2). With the exception of treatment modality, these relationships were also observed in the multivariate model when adjusting for all other covariates (Table 2).

**TABLE 2:** Predictors of a new mood‐related psychiatric disorder after diagnosis of cancer (*N* = 11,712).

Characteristic	HR^a^ (95% CI)	aHR^b^ ^,^ ^d^ (95% CI)	aES^e^
**Race**			
Non‐Hispanic (NH) White	1.00	1.00	
Asian/Pacific Islander	0.58 (0.45, 0.76)*	0.60 (0.46, 0.65)*	0.21
Other^c^	0.97 (0.89, 1.05)	0.95 (0.87, 1.03)	0.03
**Age at diagnosis (years)**			
< 50	1.00	1.00	
51–64	0.81 (0.74, 0.89)*	0.83 (0.76, 0.92)*	0.10
> 65	0.66 (0.60, 0.73)*	0.70 (0.63, 0.78)*	0.18
**Sex**			
Male	1.00	1.00	
Female	1.60 (1.48, 1.72)*	1.78 (1.64, 1.94)*	0.25
**Sexual orientation**			
Heterosexual/Straight	1.00	1.00	
Homosexual (Lesbian/Gay)/Bisexual	1.76 (1.39, 2.24)*	1.78 (1.40, 2.27)*	0.38
Other/Unknown/Undisclosed	0.59 (0.54, 0.64)*	0.59 (0.54, 0.65)*	0.24
**Tobacco use**			
Never smoker	1.00	1.00	
Former smoker	1.31 (1.21, 1.42)*	1.40 (1.30, 1.52)*	0.16
Current smoker	1.78 (1.60, 1.98)*	1.79 (1.60, 2.00)*	0.32
**Human papillomavirus (HPV) status**			
Non‐HPV associated	1.00	1.00	
HPV Associated	1.54 (1.28, 1.85)*	1.23 (1.02, 1.49)*	0.12
**Stage at diagnosis**			
0	1.00	1.00	
I	1.08 (0.93, 1.25)	1.18 (1.02, 1.37)*	0.08
II	0.97 (0.84, 1.13)	1.27 (1.09, 1.48)*	0.11
III	1.28 (1.10, 1.49)*	1.58 (1.34, 1.85)*	0.22
IV	1.85 (1.58, 2.18)*	2.31 (1.96, 2.73)*	0.43
**Treatment modalities**			
Diagnostic biopsy only	1.00	1.00	
Biopsy + hormonal monotherapy	1.29 (0.83, 2.02)	0.91 (0.58, 1.43)	0.04
All other treatment modalities	1.74 (1.40, 2.17)*	1.08 (0.86, 1.36)	0.03

^a^ HR, hazards ratio.

^b^ aHR, adjusted hazards ratio.

^c^ Other race included NH Black, Hispanic (all Races), and Multiple Races.

^d^ Adjusted for all other variables in table.

^e^ aES, adjusted effect sizes.

*p ≤ 0.05.

**FIGURE 1:**
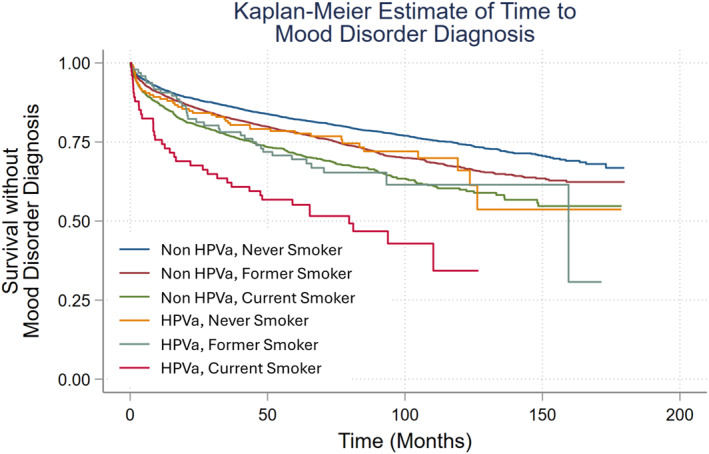
Kaplan–Meier estimate of time to mood related psychiatric disorder diagnosis post‐cancer diagnosis. HPVa = Human papillomavirus associated.

Time‐to‐MRPD differences were observed when adjusting for all other covariates and stratifying by the individual behavioral risk factors (tobacco use history and HPV‐associated cancer) (Figure 1). Shorter time‐to‐MRPD were observed among never smokers with HPV‐associated cancers relative to never smokers with non‐HPV associated cancers (Figure 1). Similarly, shorter time‐to‐MRPD were observed among current smokers with HPV‐associated cancers relative to current smokers with non‐HPV associated cancers (Figure 1). Current smokers with HPV‐associated cancers were the subgroup with the shortest time‐to‐MRPD (Figure 1).

## 4 Discussion

The findings from this study highlight several noteworthy associations. First, female sex, younger age at diagnosis (age < 50), and advanced disease stage (III or IV) were all independently associated with a moderately higher risk of new MRPD. This is consistent with prior work that has demonstrated similar relationships among these subgroups [29, 30]. Second, individuals that identify as a sexual minority were observed to have a small increased risk of a new MRPD post‐cancer diagnosis relative to those that identified as heterosexual, with a small effect size adjusted to the study sample. This finding is similar to previous research that has highlighted how sexual minorities diagnosed with particular cancer types experience higher rates of depressive and anxiety symptoms post‐cancer diagnosis [31, 32, 33]. This finding should additionally be interpreted cautiously when considering that only a small proportion of the entire study sample (*n* = 154, 1.3%) was comprised of individuals that self‐reported being a sexual minority. Additionally, 32% (*n* = 3757) of the study sample did not have a documented sexual preference which could bias our study's findings. Third, individuals that identify as Asian/Pacific Islanders were observed to have a moderately lower risk in developing new MRPD post‐cancer diagnosis, which has been observed among certain subgroups within this demographic group such as females and those with higher levels of acculturation [34, 35]. This finding however does contrast with previous research assessing comorbidity of mental and psychiatric disorders post‐cancer diagnosis particularly in the context of the United States where more anxiety and depressive symptoms have been observed [35, 36]. This may represent a situation where this demographic may experience higher mood related symptoms post‐diagnosis that do not ultimately progress to a clinically significant MRPD.

Differences in time‐to‐MRPD were observed by individual behavioral risk factors. Similar to previous research, individuals that were either former or current smokers at the time of cancer diagnosis had higher hazards of a new MRPD diagnosis post‐cancer diagnosis, with current smokers having aHRs associated with moderate effect sizes when adjusting for the study sample [9, 37, 38]. Patients with HPV‐associated cancers were also observed to have a 23% higher hazards of a new MRPD diagnosis post‐cancer diagnosis relative to those with non‐HPV associated cancers. The effect size of this difference was observed to be small (Table 2). Some studies have reported that HPV positivity to be associated with higher anxiety and depressive symptoms around the time of the initial cancer diagnosis [15, 39]. HPV association was not observed to be related to reported anxiety and depressive symptoms in the post‐treatment time period among small samples of patients with oropharyngeal or cervical cancers [15, 18]. Our study findings contrast with those previously reported in that we observed clinically meaningful diagnoses of MRPD post‐cancer diagnosis versus symptomatic monitoring of anxiety and depressive symptoms at different time points during a patient's cancer care. Our study sample additionally included patients with different types of HPV‐associated cancers (oropharyngeal, anorectal, cervical, vaginal, vulvar, and penile) versus just studying one particular disease group. Lastly, the combination of individual behavioral risk factors were observed to be predictive of shorter time‐to‐MRPD, which to the best of our knowledge has not been previously reported. Patients with more common cancers (namely those in the control group: prostate, breast and colorectal) generally have access to a wider range of psychosocial support in the form of cancer survivor support groups relative to patients with HPV‐associated cancers, which are less common. Some of the differences observed in the level of risk of developing a new MRPD may be explained by the differences in social support.

### 4.1 Study Limitations

The present study has several limitations. First, it was conducted within a single NCI‐Designated Comprehensive Cancer Center, whose results may not be generalized to community settings. Notably, the demographics (e.g. sex, age, sexual preference, smoking prevalence, etc.) of our study sample may vary from those of other geographic areas of the country. However, this study included multiple cancer types and encompassed racial and ethnically diverse cohorts. Second, although the sample size during the study period was robust, a small proportion of the sample included those diagnosed with an HPV‐associated cancer. It should also be acknowledged that the results from this study should be cautiously interpreted when considering that the sample size was powered to detect small effect differences between subgroups. Additionally, we did not molecularly determine HPV status but based this on supposition that cancers at particular anatomic sites and with specific histologies are predominantly found to be virally associated [25]. Future work should include larger samples of patients with these cancers and should be conducted with the collaboration of cancer centers that service different parts of the country to diversify the study cohort. Third, the present study employed a complete case analysis whereby cases with missing data were excluded (e.g. smoking status being assessed within 30 days of cancer diagnosis). This may introduce a selection bias in the population under study and the results. We acknowledge that the approach taken to select the comparison group could be a concern since some patients with those cancers could perceive their diagnosis to be associated with tobacco use if they had a history of smoking. Future prospective work should assess the relationship between MRPD and patient perceptions regarding the cause of their cancers. Additionally, patients with prior histories of a MRPD were excluded from the analyses as a way of assessing the potential role of individual risk behaviors on the development of a new MRPD. When considering that previously diagnosed MRPDs are a known risk factor for subsequently developing another MRPD, future work should additionally include these patients to assess their level of risk [40]. Lastly, our study did not control for access to psychosocial support which could be an important mitigator of a new MRPD post‐cancer diagnosis.

### 4.2 Clinical Implications

Overall, the present study results offer several important implications. These findings suggest that the individual behavioral risk factors of tobacco use and, to a lesser extent, HPV, are independently associated with a new MRPD post‐cancer diagnosis. While many factors likely effect risk for MRPD post‐cancer diagnosis, these findings suggest that these individual risk factors may be factors that clinicians would want to be aware of in monitoring patients for potential MRPDs and consider for support in order to maximize clinical outcomes and quality of life.

## 5 Conclusions

Prior work has demonstrated that comorbid anxiety and depressive disorders at the time of cancer diagnoses portend decreased survival relative to those without these comorbidities [24]. But, our results may provide a framework for identifying at‐risk patients using data available from the electronic health record rather than involving the use of additional clinical measures. Taken together with the findings from the present study, future work should assess whether identifying patients using such approaches earlier on can reduce risk for adverse psychiatric outcomes and improve clinical outcomes such as overall survival. Additionally, future work should assess whether treating new MRPDs after cancer diagnosis improves clinical outcomes like overall survival.

## Ethics Statement

This study was approved by the Institutional Review Board at the University of Pennsylvania (Protocol #: 853861) as an exempt study.

## Conflicts of Interest

The authors declare no conflicts of interest.

## Data Availability

The data that support the findings of this study are available from the corresponding author upon reasonable request.
